# Comparative study of machine learning and statistical survival models for enhancing cervical cancer prognosis and risk factor assessment using SEER data

**DOI:** 10.1038/s41598-024-72790-5

**Published:** 2024-09-27

**Authors:** Anjana Eledath Kolasseri, Venkataramana B

**Affiliations:** Department of Mathematics, School of Advanced Sciences, Vellore Institute of Technology, Vellore, Tamil Nadu India

**Keywords:** Cervical cancer, Survival analysis, Machine learning, Statistical methods, Prognostic factors, Cancer, Computational biology and bioinformatics, Health care, Medical research, Risk factors

## Abstract

Cervical cancer is a common malignant tumor of the female reproductive system and the leading cause of death among women worldwide. The survival prediction method can be used to effectively analyze the time to event, which is essential in any clinical study. This study aims to bridge the gap between traditional statistical methods and machine learning in survival analysis by revealing which techniques are most effective in predicting survival, with a particular emphasis on improving prediction accuracy and identifying key risk factors for cervical cancer. Women with cervical cancer diagnosed between 2013 and 2015 were included in our study using data from the Surveillance, Epidemiology, and End Results (SEER) database. Using this dataset, the study assesses the performance of Weibull, Cox proportional hazards models, and Random Survival Forests in terms of predictive accuracy and risk factor identification. The findings reveal that machine learning models, particularly Random Survival Forests (RSF), outperform traditional statistical methods in both predictive accuracy and the discernment of crucial prognostic factors, underscoring the advantages of machine learning in handling complex survival data. However, for a survival dataset with a small number of predictors, statistical models should be used first. The study finds that RSF models enhance survival analysis with more accurate predictions and insights into survival risk factors but highlights the need for larger datasets and further research on model interpretability and clinical applicability.

## Introduction

Cervix uterine cancer (CC) is the fourth leading cause of cancer-related deaths in women worldwide^1^. According to the GLOBOCAN 2020 study, over 600,000 new cases of CC were reported, with an estimated 340,000 deaths in 2020^2^. Despite extensive research, its incidence and mortality rates have been on the rise in recent years^2^. Although the World Health Organization launched the “Global strategy to accelerate the elimination of cervical cancer” in 2018, less than 30% of lower- or middle-income countries had included the human papillomavirus (HPV) vaccine in their national immunization schedules by 2020^3^. As a result, combating cervical cancer will be difficult in the coming decades.

Cervical cancer has a better prognosis than other types of malignant tumors. According to SEER statistics from 2011 to 2017, the overall 5-year survival rate for cervical cancer was 66.3%^4^. Although clinical stages are excellent predictors of cervical cancer prognosis, age, histological type, and tumor differentiation all have a significant impact^5,6^. Advanced survival analysis in cervical cancer is critical for improving patient outcomes by accurately predicting disease progression and designing personalized treatment plans. Researchers can use traditional statistical methods and machine learning techniques to identify critical prognostic factors, improve early detection, and optimize therapeutic strategies, resulting in better management of cervical cancer and survival rates. Traditional statistical methods have long been employed in survival analysis to evaluate these prognostic factors and predict outcomes. However, the advent of machine learning (ML) techniques has introduced new paradigms in predictive analytics, offering potentially more nuanced insights into survival prediction and risk factor evaluation^7^.

Nowadays, survival analysis is used in almost every scientific field, including medicine. Survival analysis is a technique that is used to determine the likelihood that a patient will experience a specific drug or procedure-related event, such as failure or death, after receiving treatment^8^. The response variable in survival data is the duration of time that remains until a particular event of interest occurs; which is the survival time or death time. Patient survival data is usually analyzed using survival models. Statistical models such as the Cox proportional hazards model and Kaplan-Meier estimator have been foundational in medical survival analysis, valued for their interpretability and extensive clinical validation^9^. However, these models often may not capture the complexities inherent in medical data^10^. Machine learning models, including Random Survival Forests, Survival Support Vector Machines, and deep learning techniques, offer more flexibility in modeling non-linear patterns and complex interactions^11^. Despite their potential for greater accuracy, ML models can be less interpretable due to their complexity and the intricate relationships between numerous parameters, making it difficult to understand and explain how predictions are derived, which is an important consideration in clinical applications^12^.

The primary goal of this study is to compare the Cox proportional hazards model, Weibull model, and Random Survival Forest (RSF) model for survival analysis in cervical cancer patients. We aim to provide insights into the strengths and limitations of each model, helping clinicians and researchers choose appropriate analytical tools based on model characteristics and assumptions. By comparing the Survival curve, error rate, Concordance index (C-index), and Integrated Brier Score (IBS), we evaluate each model’s predictive accuracy and ability to identify significant variables for cervical cancer survival prediction. This study seeks to determine if ML models offer substantial improvements over conventional statistical methods in predictive accuracy and risk factor identification. A significant aspect of this research involves assessing the impact of various clinical and demographic variables on patient outcomes, utilizing the strengths of both statistical and ML approaches^13^.

This research paper presents a comprehensive comparison between these two paradigms, using a detailed dataset on cervical cancer patients that includes variables such as stage of cancer, treatment received, and various demographic factors. By applying both traditional and modern analytical techniques to the same dataset, this study aims to provide insights into which methods are most effective in predicting survival and identifying key risk factors in cervical cancer. This comparison is expected to guide clinicians and researchers in selecting the most appropriate analytical tools for survival analysis in oncology and improve the understanding of cervical cancer prognosis. Researchers can apply the methodology through this study, which makes it easier to analyze survival data by selecting the best model and, consequently, producing the best prediction.

## Materials and methods

### Data sources and inclusion criteria

This study utilized the nationwide SEER database, which has been maintained by the National Cancer Institute in the United States since 1975, and is one of the largest and highest-quality cohort studies. It is the most widely used Medicare database for cancer datasets. This study first included female patients diagnosed with cervical cancer between 2013 and 2015. By selecting a slightly older cohort, we ensured that the data had been thoroughly vetted and that all pertinent follow-up information was available. This helps to reduce potential biases and inaccuracies that may arise when using more recent, but possibly incomplete, data. Furthermore, limiting the cohort to a specific timeframe promotes consistency in treatment practices and diagnostic criteria, which can change over time. This makes the results more comparable and reliable over the chosen time period.

Patients with cervical cancer were identified when the “Site recode ICD O3.WHO 2008” field was filled in with “Cervix Uteri”. However, some SEER patients did not receive the recommended therapies due to patient preferences or comorbidities, which may have shortened their survival and reduced the model’s performance. According to the National Comprehensive Cancer Network guidelines, cervical cancer patients should have surgery (local tumor excision such as cone biopsy, hysterectomy, modified hysterectomy, and radical hysterectomy) or radiotherapy, depending on their clinical stage. Taking the treatment-related variables in SEER into account, patients who received surgery or radiotherapy via SEER were classified as having standard treatment.

#### Data preprocessing and selection

Variables for each CC (Cervical Cancer) patient was collected and analyzed, including age at diagnosis, marital status, race, tumor stage, use of radiotherapy and chemotherapy, tumor size, TNM staging, household income, survival months, and vital status(alive/dead). Several columns in the selected instances were missing values. Missing values were replaced with the mean or mode based on the attribute type, and a few rows with a high number of missing values were removed. After preprocessing, the cervical cancer dataset contains 3810 instances. The selected instances were stored as CSV files. The data instances are separated into training and test data sets for training and evaluating prediction models.

The “Survival months” attribute, a continuous variable that indicates the number of months a patient survived from the date of diagnosis, represents the primary outcome of interest in this study: the patient’s survival time. When determining the patient’s survival status at the conclusion of the follow-up period and identifying censored data, the “Vital status” attribute—a binary variable that indicates whether the patient is alive (0) or dead (1)—is used.

### Methods

#### Semi-parametric survival models

The Cox regression model is a semi-parametric survival model that does not require a fixed distribution for the baseline hazard, making it more flexible than fully parametric survival models. It combines parametric and nonparametric components, with a focus on estimating covariate hazard ratios^14^. The Methodology for Estimating the baseline hazard function includes^15^:

- **Partial Likelihood**: The Cox model first estimates the regression coefficients (β) of the covariates using partial likelihood, which does not require the baseline hazard $\:{h}_{0}\left(t\right)$ to be specified. This method leverages the ordering of events to estimate the effects of covariates without explicitly estimating $\:{h}_{o}\left(t\right)$.
- **Baseline Hazard Estimation**: After estimating the coefficients β, the baseline hazard function $\:{h}_{0}\left(t\right)$ is derived using the Breslow estimator, which provides a step function estimate of the cumulative baseline hazard $\:{H}_{0}\left(t\right)$. The baseline hazard at each event time $\:{t}_{j}$ is estimated as:
  $${H_0}\left( {{t_j}} \right)=\mathop \sum \limits_{{i \leqslant j}} \frac{{{d_i}}}{{\mathop \sum \nolimits_{{l \in R\left( {{t_i}} \right)}} {\text{exp}}\left( {{x_l}\beta } \right)}}$$
  where $\:{d}_{i}$ is the number of events at time $\:{t}_{i}$ and $\:{R}_{i}$ is the risk set at time $\:{t}_{i}$.
- **Hazard Function**: The hazard function $\:h\left(t|X\right)$ is then obtained by differentiating the cumulative hazard function $\:{H}_{0}\left(t\right)$ with respect to time. The hazard rate $\:h\left(t|X\right)$ is assumed to be equal to a baseline hazard $\:{h}_{0}($t) modified by p covariates X = ($\:{X}_{1},{X}_{2},.{X}_{p}$) and their respective coefficients β = ($\:{\beta\:}_{1},{\beta\:}_{2},\dots\:{\beta\:}_{p})$:
  $$h\left( {t{\text{|}}X} \right)={h_0}\left( t \right){e^{{\beta ^T}X}}$$

Usually, the primary interest lies in estimating the coefficients β, as the baseline hazard $\:{h}_{0\left(t\right)}$ is considered a nuisance parameter^16,9^. The estimation of $\:{h}_{0}(t$ ) is done after the covariate effects have been determined using Cox’s partial likelihood.

#### Parametric survival models

The hazard rate in parametric survival models is typically assumed to have some shape (flat, monotonic, etc.). One advantage of using a parametric distribution is that it is possible to predict the time to event for the observed data well after the period in which events occurred^17^. Among the popular parametric survival regression models, the simple parametric model, and the Weibull model of regression were considered in this study. In the Weibull model, the baseline hazard function $\:{h}_{0}\left(t\right)$ is assumed to follow a Weibull distribution, defined as:

$${h_0}\left( {t{\text{|}}\lambda ,\alpha } \right)=\alpha {\lambda ^\alpha }{t^{\alpha - 1}}:\left( {\lambda ,\alpha >0);~t \geqslant 0} \right)$$

It has two positive real parameters λ and α, to be estimated from a training set.

When covariates X = ($\:{X}_{1},{X}_{2,}\dots\:,{X}_{p}$) are included, the hazard function $\:h(t|X)\:$is expressed as:

$$h(t|X)=\alpha\lambda^\alpha {t^{\alpha - 1}}{\text{exp}}\left( {{\beta ^T}X} \right):\left( {\lambda ,\alpha >0);~t \geqslant 0} \right)$$

where $\:\beta\:$ is the vector of coefficients for the covariates. This formulation allows the model to account for individual differences in risk based on their covariate values.

Key differences between semi-parametric and parametric models:

- In parametric models like the Weibull model, the baseline hazard function is specified as a parametric function (e.g., $\:{h}_{0}\left(t\right)$= $\:\lambda\:\alpha\:{t}^{\alpha\:-1}$ for the Weibull distribution). This allows for direct estimation of the baseline hazard parameters^18^.
- In the semi-parametric Cox model, the baseline hazard function is not specified in advance, providing more flexibility and reducing the risk of model misspecification. However, this also means that $\:{h}_{0}\left(t\right)$ must be estimated non-parametrically after the covariate effects are determined^19^.

#### Random forests for survival analysis

Random Survival Forests (RSFs) are an ensemble tree method for survival analysis of right-censored data, derived from random forests^20^. Here, Random survival forests were chosen because they can handle high-dimensional data, capture complex interactions between variables, and are resistant to overfitting, all while providing variable importance measures that aid in interpreting the results, outperforming other machine learning survival techniques^21^. Random forests use decision trees, which have high variance but can capture complex interactions, to average their characteristics. This approach transforms weak individual trees into strong ensembles^22^.

Randomness is introduced into RSFs in two ways: by bootstrapping several patients at each tree B times and selecting a subset of variables to grow each node. To grow a survival tree, binary splitting is applied recursively to each region (a node) on a specific predictor to maximize survival differences between daughter nodes while minimizing differences within them. Splitting ends when a specific criterion is met, referred to as terminal nodes. The log-rank test by Segal^23^ and the log-rank score test by Hothorn and Lausen are the most widely used splitting criteria^24^. Each terminal node should have at least a set number of unique events.

Survival trees are based on the principle of event conservation. Ensemble mortality is a predicted outcome for survival data based on the ensemble cumulative hazard function, similar to the Cox model’s prognostic index. This principle asserts that the sum of estimated cumulative hazard estimates over time is equal to the total number of deaths, therefore the total number of deaths is conserved within each terminal node^11^. RSFs are capable of handling both large sample sizes and a large number of predictors. They assume no particular functional form and treats outcomes as binary, dividing data into high and low survival groups based on features. This allows for complex interactions. Furthermore, they can achieve remarkable stability by combining the results of multiple trees. Combining multiple trees significantly reduces their intuitive interpretation compared to a single tree.

#### **Model training**

The split sample approach was used, dividing data randomly into two parts: a training set (2/3) and a test set (1/3) with equal event/censoring proportions. To fine-tune a model, 5-fold cross-validation was applied in the training set for the Random survival Forest. It is a common and recommended practice in the machine learning community. A 5-fold cross-validation enables fair comparisons of different models and methodologies while striking a reasonable balance between computational efficiency and performance estimation accuracy^25^.

Training data was divided into five folds. Four folds were used to train a model, followed by the remaining fold to validate its performance. This process was repeated for all fold combinations. The hyper-parameters were tuned through grid search, and the final models’ performance was evaluated on a test set. Analyses were carried out using R 3.5.3. and python.

#### Assessing predictive performance on test data

The survival curve, the concordance index, the Integrated Brier Score (IBS), and the error rate evaluate the models’ predictive performance. Survival curves are important for visualizing the probability of survival over time, allowing for a direct comparison of different groups’ survival experiences or the predictive performance of various models. We used the log-rank test to compare the survival curves of the Weibull, Cox, and Random Survival Forest (RSF) models against the observed survival data. A low p-value (< 0.05) indicates a statistically significant difference between the survival curves, implying that the models predict survival differently. This method is especially useful for determining whether one model has significantly better predictions of survival outcomes than others.

The concordance index is a popular measure of model performance in survival contexts. The models’ ability to discriminate was assessed using the concordance index (C-index). A higher C-index denotes better model discrimination. It evaluates the model’s accuracy in ranking the survival times according to predicted risk scores. A model’s ability to accurately distinguish between people who have different outcomes is referred to as its discrimination ability. It shows how well the model can distinguish patients with varying survival times based on their estimated risk scores in the context of survival analysis. The model’s discrimination ability is measured on a scale of 0.5 to 1, with higher values indicating greater ability and 0.5 indicating no discrimination. The concordance index provides a time-independent rank statistic for observations^26^.

The Integrated Brier Score (IBS) is a comprehensive metric for determining the accuracy of probabilistic forecasts in binary and multi-class classification models. As an extension of the Brier Score, the IBS calculates the mean squared difference between predicted probabilities and actual outcomes, combining calibration and sharpness into a single score^27^. Specifically, the Brier Score for binary classifications is calculated as,

$${\text{BS }}=\frac{1}{N}\sum\nolimits_{{i=1}}^{N} {{{({p_i} - {o_i})}^2}}$$

where *N* is the number of predictions, $\:{p}_{i}$ is the predicted probability of the positive outcome for instance *i*, and $\:{o}_{i}$ is the actual binary outcome.

The Integrated Brier Score averages these measures across multiple time points or forecasts, providing a useful summary of a model’s predictive performance by capturing both its reliability in probability estimates and its ability to confidently discriminate between classes^28^.

Additionally, the models’ performance was evaluated using the error rate, which is the percentage of incorrect predictions the model made. Improved model accuracy is indicated by a lower error rate.

#### Interpretability of the models

Interpreting models is important for the medical community. Survival models such as the Weibull model, the Cox proportional hazards model, and Random Survival Forests all provide different approaches to analyzing time-to-event data, with varying degrees of interpretability. The Weibull model is valued for its parametric simplicity and clear interpretation via its scale and shape parameters, which represent the hazard rate’s time scale and trend, respectively^29^. In contrast, the Cox proportional hazards model, a semi-parametric approach, allows for estimating hazard ratios without specifying the baseline hazard function, making it highly interpretable regarding how covariates affect the hazard, subject to the proportional hazard’s assumptions^9^. Here, the Weibull model and the Cox proportional hazards model are examined separately. Taking advantage of the semi-parametric nature of the Cox model, it was applied without defining the baseline hazard function h(t). On the other hand, the Weibull model was considered as a parametric model, where h(t) was subject to the Weibull distribution. As an alternative, we decided to analyze each model independently to emphasize its unique strengths. It is possible to combine these models by using a Weibull distribution as the baseline hazard in the Cox framework, which allows for simultaneous estimation of Weibull parameters and Cox coefficients.

On the other hand, Random Survival Forests take a non-parametric approach, dealing with complex interactions and non-linear relationships without making predefined assumptions about data structure, but this comes at the expense of straightforward interpretability due to their ensemble nature and decision tree complexity^21^. While RSFs are useful tools for assessing variable importance and partial dependence, their overall complexity and the nature of ensemble methods make them less interpretable than simpler models. Efforts to improve the interpretability of RSFs, such as visualizing decision trees and employing supplementary interpretability techniques, can help bridge this gap, but they require more effort and expertise^11,30^.

Thus, while the Weibull and Cox models provide more direct insights into the effects of covariates on survival times, Random Survival Forests provide robust predictive capabilities while taking a nuanced approach to interpreting variable importance and effects on survival outcomes.

## Results

### Selection of patient attributes

Table 1 shows a list of 13 key attributes and their encoding method, including the target “Survival months,” which include both continuous (numeric) and categorical (discrete) variable types. The models were then used to correlate the remaining 12 attributes with patient outcomes, specifically survival time. The database contains data on patients diagnosed with cervical cancer between 2013 and 2015. The survival time range is 0–95 months, and the models’ output is expected to fall within this range.

**Table 1:** Selected SEER attributes and their respective descriptors and encoding methods.

No.	Attribute	Description	Type	Encoding method (Cox & Weibull)	Encoding method (RSF)
1	Age	Age at time of diagnosis.	Numeric	Continuous	Continuous
2	Stage	Stage of tumor – based on T, N, and M.	Categorical	Dummy coding for each stage	Handled directly by RSF (no explicit coding needed)
3	T stage	AJCC component describing tumor size.	Categorical	Dummy coding for each category	Handled directly by RSF
4	N stage	AJCC component describing lymph node involvement.	Categorical	Dummy coding for each category	Handled directly by RSF
5	M stage	AJCC component describing tumor dissemination to other organs	Categorical	Dummy coding for each category	Handled directly by RSF
6	Radiation therapy	Indication of whether the patient has received radiation therapy	Categorical	0 (no), 1 (yes)	0 (no), 1 (yes)
7	Chemo therapy	Indication of whether the patient has received chemotherapy	Categorical	0 (no), 1 (yes)	0 (no), 1 (yes)
8	Race	Race of the individual	Categorical	Dummy coding for each category	Handled directly by RSF
9	Marital status	Indication of the marital status of the individual	Categorical	Dummy coding for each category	Handled directly by RSF
10	Household income	Indication of household income of the individual	Categorical	Dummy coding for each income	Handled directly by RSF
11	Tumor size	Measurement of tumor size.	Numeric	Continuous	Continuous
12	Vital status	Indication of whether the patient is alive or dead	Categorical	0(alive),1(dead)	0(alive),1 (dead)
13	Survival months	Number of months that patient is alive from date of diagnosis.	Numeric	Continuous	Continuous

AJCC: American Joint Committee on Cancer.

### Distribution of survival times

**Fig. 1:**
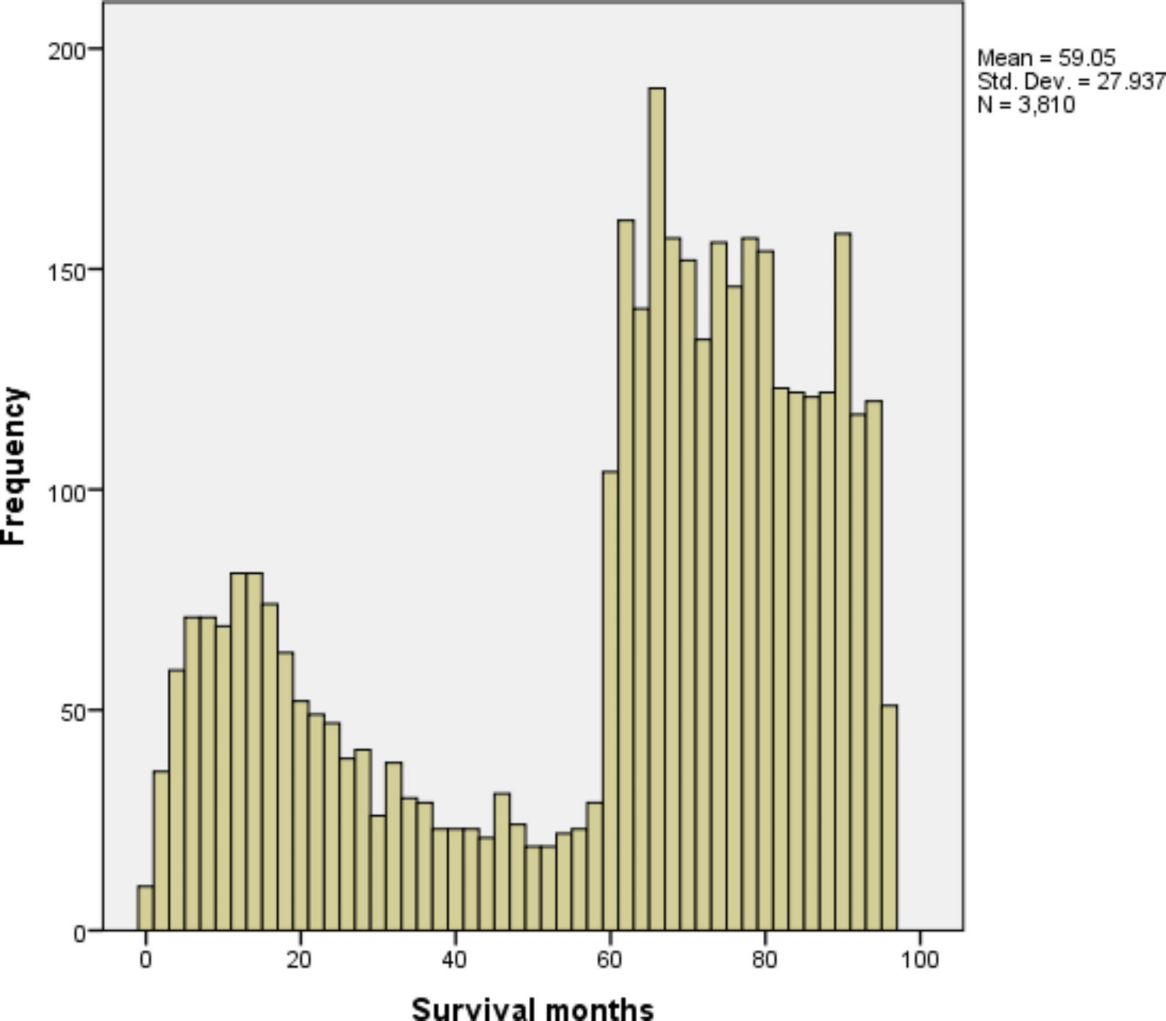
Survival time (months) for cervical cancer patients in the SEER database (2013–2015).

The dataset retrieved from the SEER database included 3810 patient records after preprocessing. Figure 1 illustrates the overall distribution of survival times as a histogram, which is useful for demonstrating the distribution of a continuous variable, such as survival time, as well as how frequently each range of values appears^31^. Across the entire set, the average survival time is 59.05 months with a standard deviation of 27.93.

### Comparisons between models

**Fig. 2:**
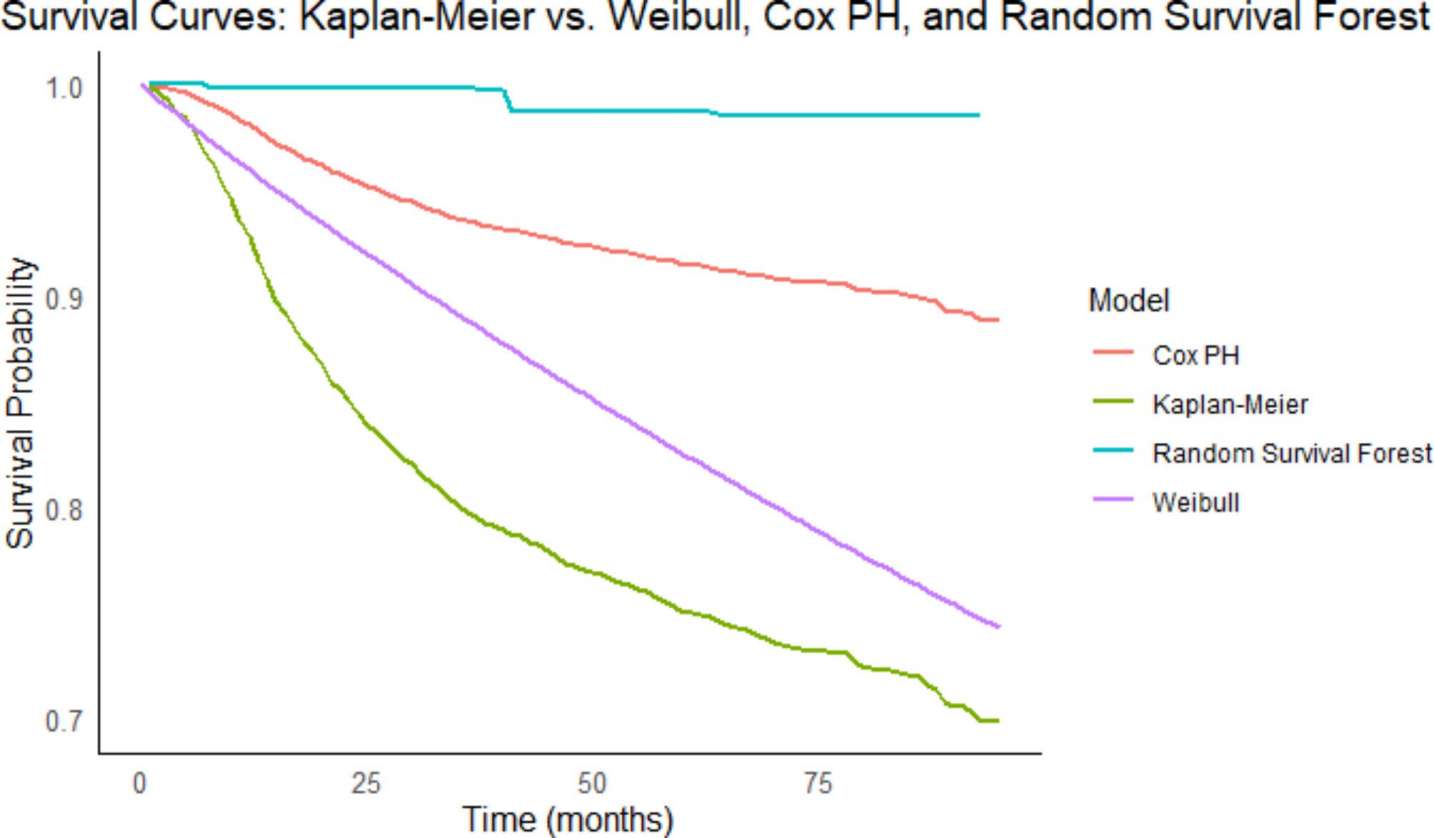
Survival curves of Cox Proportional Hazards (Cox PH), Weibull, and Random Survival Forest (RSF) models compared against the observed Kaplan-Meier survival curve.

After arranging attributes, data was analyzed using statistical and machine learning techniques. To evaluate the predictive performance of the Cox proportional hazards, Weibull, and Random Survival Forest (RSF) models, we created survival curves for each model and compared them to the actual observed Kaplan-Meier survival curve. The survival curves were plotted on the same graph to allow for direct visual comparison (Fig. 2).

The survival curves of each model were closely examined to ensure that they matched the observed data. We used a log-rank test to statistically compare the survival curves. The log-rank test yielded a p-value of one, indicating that there were no statistically significant differences between the survival curves generated by the Cox, Weibull, and RSF models and those observed. This suggests that while all models provide plausible estimates of survival, they each have different characteristics and implications. The Weibull model appears to be the most conservative, while the Cox PH model is more optimistic. The RSF model is unique in its flat initial curve, which could indicate it captures different aspects of the data or possibly overfits. Further validation and analysis would be required to determine the most appropriate model for the data. Therefore, other metrices such as C-index, IBS and error rate to compare these 3 models were used. Table 2 shows the performance (measured using the concordance index), the error rate, and the Integrated Brier Score (IBS). This section shows a direct comparison of the three models, and important variables are identified using a variable importance plot. Dummy coding was used to specify related variables, which included socio-demographic factors.

**Fig. 3:**
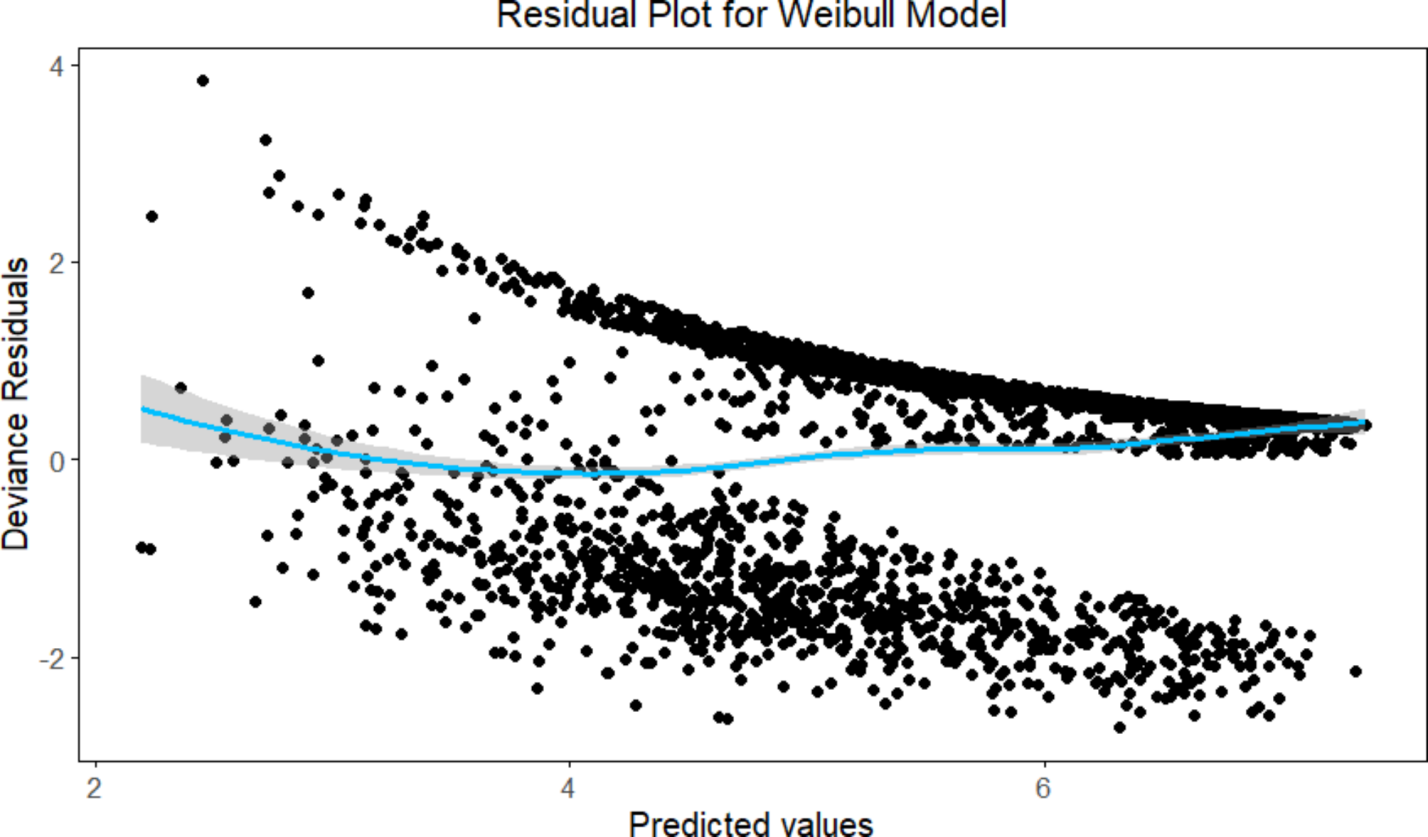
Variable Importance plot for (**a**) Weibull model (**b**) Cox- Proportional model (**c**) Random Survival Forest.

Weibull regression, a parametric model, assumes a specific distribution (Weibull) for survival times, allowing for extrapolation beyond observed data and providing a more accurate fit under certain conditions^18^. On the other hand, the Cox model assumes that all covariates have a multiplicative effect on the hazard function, which remains constant over time. Including more prognostic factors in a model can violate the proportional hazards assumption for certain covariates^32^. Clinicians can still benefit from knowing the hazard ratios for risk factors, representing the average effect on outcomes. Including all non-linear effects on interactions creates a complex model with high parameter estimates and limited interpretability. ML techniques perform well even when PH is violated because they do not rely on data structure assumptions. The variable’s importance for all the models is provided in Fig. 3. The plots visually represent the importance of each variable in the models, with higher bars indicating greater importance.

**Fig. 4:**
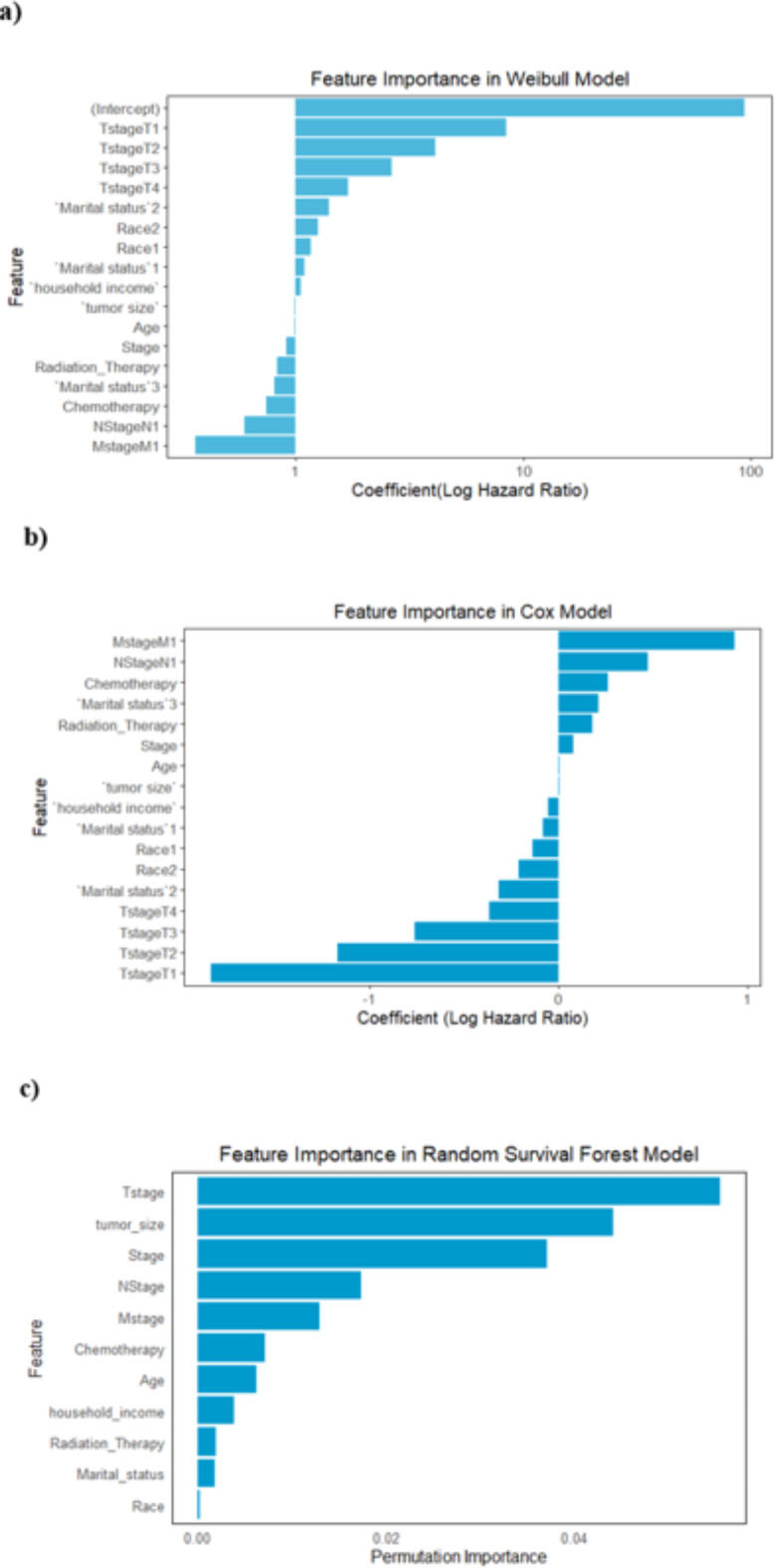
Residual plot for the Weibull model.

To account for the varying effects of different tumor stages on survival, the Weibull and Cox proportional hazards models divide the T stage into four categories (T1 to T4). This categorical representation enables these models to calculate separate hazard ratios for each stage, indicating their distinct prognostic significance^16^. Also, both models identified Tstage as a significant factor, but the coefficients had different signs. This discrepancy is due to differences in how each model estimates the hazard function and the impact of covariates. The intercept value in the Weibull model plot represents the baseline hazard rate, which is influenced by the model’s scale and shape parameters, making it difficult to interpret. Depending on the values of these parameters, it is assumed that the hazard is either increasing, continuing the same, or decreasing over time. The discrepancy may arise from the Weibull model’s assumptions not being appropriate for the dataset used in this study. For example, inaccurate parameter estimates, including the sign of the coefficients, may result if the true underlying hazard function deviates from the particular form assumed by the Weibull model (for example, if the hazard is not proportional or does not follow a monotonic pattern). To investigate this, we ran a goodness-of-fit test on the Weibull model and looked at the residual plots (Fig. 4). The results showed a poor fit, implying that the Weibull model may not accurately represent the relationship between predictors and survival time in this dataset, because the presence of a non-random pattern and variation in the spread of residuals across different levels of predicted values indicates potential issues with the model’s assumptions and ability to accurately capture the relationship between the predictors and the outcome. Specifically, the discrepancy in the T-stage coefficient between the Weibull and Cox models may be due to the parametric nature of the Weibull model, which assumes a specific form for the baseline hazard function. This can lead to differences in how the hazard is estimated across different T-stage levels if the assumed hazard function does not align well with the data. The Cox model, on the other hand, being semiparametric does not rely on a specific baseline hazard function, which may make it more robust in situations where the true hazard function is complex or unknown.

The RSF model, as a non-parametric ensemble method, treats categorical variables differently. It does not require the same level of detail as parametric or semi-parametric models to make accurate predictions. Instead, the RSF model can capture complex interactions and nonlinear relationships between variables, even if they are not explicitly classified into multiple categories^21^, as shown in Fig. 3c. In our study, the RSF model was designed to use the entire T stage as a single feature, simplifying the model and improving interpretability. This approach still captures the influence of the T stage on survival outcomes, but it does so in a way that is more appropriate for the strengths of the RSF method. It is consistent with the hierarchical structure of Random Forest and its capacity to handle categorical variables and intricate interactions.

A comparative study shows that the RSF model is preferred at capturing complex interactions with flexible feature influence, whereas traditional models like Cox provide interpretability through proportionate hazards and semi-parametric assumptions. These variations emphasize the importance it is to select a model depending on the properties of the data and the objectives of the analysis.

**Table 2:** Error rate, integrated brier score (IBS), and C-index on the data.

Model	C-index	Brier score	Error rate
Weibull model	0.80	0.04	0.006
Cox-proportional hazard model	0.81	0.03	0.006
Random survival forest method	0.95	0.02	0.0019

### Variable importance

This section compares the models based on the most significant prognostic variables among the 11 predictors. Table 3 displays the hazard ratios for the top 11 prognostic variables in both the Cox and Weibull models based on absolute z-score.

**Table 3:** Hazard ratios along with their 95% confidence intervals for the 11 most influential variables for the Weibull and Cox models.

Variables	Weibull HR (95% CI)	Cox HR (95% CI)
TstageT1	8.105 (2.384, 27.562)	6.220 (1.069, 12.704)
TstageT2	3.973 (1.172, 13.468)	4.324 (0.102, 10.032)
TstageT3	2.546 (0.753, 8.604)	3.502 (0.158, 7.595)
TstageT4	1.612 (0.468, 5.555)	1.520 (0.159, 4.705)
NStageN1	0.591 (0.502, 0.695)	1.411 (1.209, 1.647)
MstageM1	0.354 (0.230, 0.547)	1.140 (0.712, 1.826)
Stage	0.895 (0.718, 1.117)	0.830 (0.631, 1.405)
Radiation therapy	0.545 (0.350, 1.098)	0.687 (0.452, 1.385)
Chemotherapy	0.737 (0.560, 0.971)	0.627 (0.471, 0.958)
Tumor size	0.995 (0.994, 0.997)	1.004 (1.003, 1.005)
Age	0.989 (0.980, 0.997)	1.007 (0.999, 1.016)
Race	1.118 (1.023, 1.223)	0.940 (0.778, 0.137)
Marital status	0.142 (0.063, 1.228)	0.740 (0.581, 0.941)
Household income	1.061 (1.031, 1.092)	1.261 (0.868, 1.832)

The hazard ratios (HRs) from both the Weibull and Cox models for cervical cancer survival indicate that tumor stage (TstageT1 to TstageT4) has a significant impact on survival, with early stages generally associated with better outcomes, as evidenced by the Cox model’s protective effect of lower T stages. The Weibull model suggests a protective effect for NStageN1 and certain treatments, indicating potentially effective management of early-stage diseases. However, the Cox model identifies these as negative factors, reflecting their association with advanced disease stages, which are inherently linked to poorer outcomes. Socioeconomic factors, particularly household income, are significant in both models, with the Cox model emphasizing the negative impact of lower socioeconomic status on survival, underscoring the importance of social determinants in health outcomes.

Table 4 shows the most significant prognostic factors for the random survival forest (RSF). The top predictors are listed based on their variable importance (VIMP) for the RSF. VIMP is a measure used in Random Forest models to quantify the importance of each predictor variable. It measures the increase in prediction error when the values of a particular variable are permuted while all other variables remain unchanged. Higher VIMP values indicate greater importance of the variable in the model’s predictive power. The Random Survival Forest’s variable importance plot effectively identifies key predictors of survival outcomes, demonstrating the model’s strength in capturing significant relationships even in complex datasets.

**Table 4:** VIMP values for random survival forest.

Variable	Variable importance (VIMP)
T-stage	0.055
Tumour size	0.044
Stage	0.036
N stage	0.017
M stage	0.012
Chemotherapy	0.044
Age	0.006
Household income	0.0038
Radiation therapy	0.0019
Marital status	0.0018
Race	0.0002

The feature importance graph shows that T-stage (VIMP- 0.055) and tumor size (VIMP- 0.044) are the most influential factors, with the highest permutation importance values, implying that these variables have the greatest impact on the model’s predictions. Stage (VIMP- 0.036) and N-stage (VIMP- 0.017) play an important role in survival outcomes. Other variables, such as M-stage, chemotherapy, and age, make a moderate contribution to the model’s predictions. In contrast, features such as household income, radiation therapy, marital status, and race are less important, implying a smaller role in determining survival in this dataset. Overall, the RSF model effectively highlights the key predictors of survival, emphasizing the clinical significance of tumor-related variables in a way that is simpler than the Cox and Weibull models.

## Discussion

As modern data collection techniques expand, it’s crucial to develop methods for analysing high-dimensional, heterogeneous clinical data sets. This data can be used to improve patient allocation systems, provide more accurate prognoses and diagnoses, and identify risk factors. In recent years, machine learning (ML) has gained popularity in medical area. For example, in cervical cancer, developing a miRNA-based machine learning survival prediction model can improve prognosis forecasting capability^33^. Sun GLi, Cao Y, et al. presented a framework for cervical cancer diagnosis based on a random forest (RF) classifier with relief feature selection^34^.

This study estimated statistical and machine learning models for patients using the SEER database. The comparison of survival curves, supported by log-rank test results, indicates that, while the Cox PH, Weibull, and RSF models predict survival differently, they are statistically similar to the observed data. Each model provides a distinct view of the data, with the Cox PH and Weibull models providing more traditional survival curves and the RSF model providing a unique, potentially more complex interpretation^11^ within the data that might be missed by parametric approaches. As a result, the study’s specific context and additional validation measures (For e.g.: C-index) should inform the model selection process. Regarding the C-index, random survival forests outperformed Cox and Weibull models. This demonstrates the model’s ability to distinguish between low and high-risk patient groups. Additionally, the Brier score was assessed for all methods. The RSF produced similar results to the Cox models, with a slightly lower total prediction error (in terms of IBS). RSF uses different learning methods to automatically model non-linear relationships between variables. It could be used in medical applications, but for the time being, it should only be used as an additional analysis for comparison.

The focus was on interpreting the models. An indirect comparison was conducted to determine which variables are most prognostic for a Weibull, Cox, and RSF. According to Weibull and Cox methods, T staging is the strongest predictor, followed by N Stage, age, tumor size, and household income. In this analysis, the T-stage coefficient sign differed between the Weibull and Cox models, which can be attributed to their respective assumptions. The Cox model being semi-parametric, does not make assumptions about a specific baseline hazard function^26^ and thus may better fit the actual data structure. The Weibull model’s parametric nature, which relies on scale and shape parameters, and assumes a specific hazard function, most likely misrepresented the data due to violations of its underlying assumptions, as evidenced by the poor goodness-of-fit results. This implies that the Cox model provides a more reliable and clinically relevant interpretation of T-stage effects in this context. These results emphasize how crucial it is to comprehend the underlying assumptions and implications of each model when interpreting survival analysis data. Whereas, the Random Survival Forest (RSF) model identified T-stage, tumor size, Stage, and N-stage as the most important predictors of survival outcomes. Higher T-stages indicate more advanced tumors, which leads to a worse prognosis, whereas tumor size and stage emphasize the importance of tumor progression in survival predictions. The model also highlighted the impact of chemotherapy and age, albeit to a lesser extent, implying that while these factors are important, they are not as influential as the primary tumor-related characteristics. Interestingly, socioeconomic and demographic factors such as household income, race, and marital status were found to be less important, indicating that while these factors may influence overall health outcomes, they play a smaller role in predicting survival in this patient population^35^. This finding is consistent with previous research, which has emphasized the importance of clinical factors over socio-demographic variables in cervical cancer prognosis^36^. The RSF model’s results also highlight a well-known bias toward continuous and multilevel categorical variables, which are preferred during the model’s binary partitioning due to their higher information content^21^. his bias was evident in the prominence of clinical variables over socio-demographic factors in the model’s variable importance ranking. Our findings are consistent with previous studies, but additional clinical predictors have been identified by others. Several clinical predictors were identified as significant risk factors for cervical cancer survival in the reviewed studies, following feature selection. Variables included age^37^, tumor grade^38^, tumor size^39^, lymphovascular invasion^40^, timely treatment^41^, and overweight or obesity^42^. Further research is needed to identify the most accurate and significant predictors of cervical cancer. Understanding these factors aids in the development of interventions and policies to improve survival outcomes across diverse population groups. Future research should analyze larger cervical cancer samples and incorporate the features identified in our study.

This is the first study to compare traditional statistical models with machine learning models on cervical cancer data, taking into account the global increase in cases. The Cox model has been used in numerous studies to compare results. In previous studies, Wright et al.^43^ compared the prediction ability and operation time of various models combined with examples, discovering that forest models outperformed the Cox regression model and that each forest model has advantages. Based on a large amount of case data for oropharyngeal cancer, Du et al.^44^ found that RSF outperformed the Cox regression model in terms of predictive accuracy. Similarly, RSF outperforms both of our statistical models in terms of C-index, IBS, and error rate. Compared to machine learning techniques, Cox and Weibull models provide an easy interpretation through hazard and parametric assumptions while the RSF model excels in capturing complex interactions with flexible feature influence. Random Survival Forest (RSF) do not provide explicit coefficients with signs (positive or negative effects) like traditional regression models. However, by inspecting the resulting decision trees, it is possible to determine the direction of the effect of predictor variables on survival time. Each decision tree in the forest divides the data based on predictor variables, indicating whether an increase in one variable correlates with an increase or decrease in survival time. ML techniques rely on randomization, resulting in minimal differences between iterations of a single algorithm. To assess performance stability, RSF were run multiple times using the same parameters. It remained stable after a certain number of trees. These differences highlight the importance of model choice based on data characteristics and analysis goals. However, the interpretation of statistical models and ML techniques cannot be directly compared using a common metric. Future research in this area is required.

RSF has the advantage of random forest, which can prevent model overfitting through random sampling^45^. Furthermore, RSF can handle high-dimensional data to filter variables and be applied to competing risk analysis^46^. RSF, a direct extension of the random forest, has been successfully used in a variety of clinical studies, including breast cancer, gastric cancer, and heart failure et al.^47–49^. To interpret Random Survival Forest (RSF) results for clinicians and researchers, highlight key outputs like variable importance measures and partial dependence plots to showcase significant predictors and their effects on survival outcomes, using visual aids like survival curves and cumulative hazard functions for clarity^50^. Ensure clear communication and collaboration between data scientists and clinical teams to effectively integrate these insights into clinical decision-making. Certainly, this opened up a plethora of combinations for traditional statistical methods, with many possibilities in medical research.

Interestingly, the predictive capabilities represented by the C-index in test datasets were similar between both statistical methods, Weibull and Cox, for predicting cervical cancer survival using the SEER database, implying that parametric and non-parametric methods produce similar results. Furthermore, the c-index of traditional and machine learning methods is nearly identical (not exactly). There are several possible explanations for the similar predictive performance of RSF and statistical models (Weibull and Cox). The first reason is that the study used a small number of predictors. Though no threshold number of predictors was available, more variables could help machine learning algorithms outperform traditional statistical models. Second, the predictors in the SEER program were gathered based on prior clinical knowledge, and many of the variables were primarily linearly related. Thus, although RSF is a non-parametric method, it may have captured the underlying structure of data^51^, which corresponded to the hypothesis that we made in the conventional models.

In this regard, for a given survival dataset with a small number of predictors, a Cox model should be considered first. Meanwhile, machine learning algorithms can be used in conjunction with traditional methods to extract additional information (for example, non-linear interactions and variable importance) from data that Cox models do not understand. It is also worth noting that RSF has a computational burden and cannot compete with Cox regression methods in terms of computation speed. Therefore, when choosing analytical tools for survival analysis in oncology, clinicians and researchers should take these factors into account to make sure the model they choose fits their particular data and research goals.

## Limitations and future improvements

There are several limitations to this study. The primary limitation of this study is the absence of data for other well-known predictors of cervical cancer survival in the SEER program. The SEER database does not include clinically significant variables such as patient baseline characteristics, such as human papillomavirus infection, hemoglobin levels, nutritional status, treatment duration, toxicities, and subsequent treatment modalities. Therefore, external validation data sets in a real-world setting should be used to confirm the study’s findings. Second, Data variability, including transformations and normalizations, poses a significant challenge to the widespread use of machine learning algorithms. To improve predictive models, we need to add more features for analysis. Third, the theories of various ML algorithms are obscure and complex. To be effective, clinicians require models that are both understandable and capable of estimating uncertainties^52^. Finally, the possibility of applying the SEER database results to other populations, such as those in low- and middle-income countries, must be confirmed in future studies.

## Conclusion

The medical field is increasingly recognizing the importance of machine learning (ML) techniques and their applications. The use of these algorithmic approaches can lead to the discovery of patterns in data, allowing for faster and more accurate decision-making. In the current study, we first chose 11 readily available factors, including demographic and clinical variables. Using statistical and machine-learning models, we identified three variables as important prognostic factors for cervical cancer. The variables are T staging, N stage, Tumor size, and Household income. This could be due to their direct influence on disease characteristics, access to healthcare, and underlying health disparities. Understanding these factors aids in the development of interventions and policies that improve survival outcomes for various population groups.

In this study, three parametric (Weibull), semiparametric (Cox-proportional), and machine learning methods (Random survival forest) were discussed using SEER data from 11 predictors. This comparative analysis shows that The RSF model excels at capturing complex interactions and providing flexible feature influence, making it ideal for datasets with complicated relationships. Traditional models, such as Cox, provide valuable interpretability via proportional hazards and semi-parametric assumptions, which are essential for clear and straightforward analyses. These results emphasize the significance of choosing a model according to the particular characteristics of the data and the analysis’s goals, making sure that the method of choice is in line with the objectives of the study and the characteristics of the dataset.

In summary, Statistical models are preferred for their ease of interpretation and speed of implementation. Our findings suggest that caution should be exercised when applying ML methods to survival data. Both approaches can be used for exploratory and analytical purposes, but it’s important to present their advantages and disadvantages.

## Data Availability

The datasets analyzed during the current study are available in the SEER repository, https://seer.cancer.gov/.
